# Protocol for a systematic review of the association between chronic stress during the life course and telomere length

**DOI:** 10.1186/2046-4053-3-40

**Published:** 2014-04-30

**Authors:** Jacklyn Quinlan, Mai Thanh Tu, Étienne V Langlois, Mohit Kapoor, Daniela Ziegler, Hassan Fahmi, Maria Victoria Zunzunegui

**Affiliations:** 1 Department of Medicine, Research Centre of the University of Montreal Hospital Centre (CRCHUM), 3875 St-Urbain St, Montreal, Quebec H2W 1V1, Canada; 2 School of Public Health, University of Montreal, Pavillion 7101, Parc Avenue, C.P. 6128, Succ. Centre-Ville, Montreal, Quebec H3C 3J7, Canada; 3 Documentation Center of the University of Montreal Hospital Centre (CHUM), Saint-Luc Hospital, 1058, Saint-Denis St., Principal Pavilion, 1st floor #1303, Montreal, Quebec H2X 3J4, Canada; 4 Montreal University Public Health Research Institute (IRSPUM), Pavillion 7101, Parc Avenue, C.P. 6128, Succ. Centre-Ville, Montreal, Quebec H3C 3J7, Canada

**Keywords:** stress, chronic social stress, telomere length, life course adversities, systematic review, protocol

## Abstract

**Background:**

The effects of stress on ill health have become evident in recent years. Under acute stress situations, a cascade of physiological events helps the body mount an appropriate adaptive response. However, under chronic stress situations, this physiological response may lead to wear and tear on the body that accelerates the decline in physiological functioning and increases the risk of chronic conditions. Recent evidence for social stress experienced during childhood suggests serious consequences many years later, even later life. Telomere length, a marker of cell aging, may provide a link between chronic social stress and age-associated physical and mental decline and risk of chronic conditions. This study examines whether chronic social stress is associated with telomere length throughout the life course.

**Methods/Design:**

We will perform a systematic review of the literature on the relationship between chronic social stress, for example, due to violence, extreme poverty, or caregiving of people with disabling conditions (exposure), and telomere length (outcome) by searching electronic databases in MEDLINE (PubMed interface), EMBASE (OVID interface), Cochrane Central (OVID interface) and gray literature from their start date onwards. We will limit the search to studies performed on human populations. Two reviewers will conduct standardized screening, eligibility assessment, data abstraction, and scientific quality assessment. All study designs investigating the association between chronic social stress and telomere length in healthy or diseased adults and children will be eligible for inclusion in the review. We will extract individual demographic and socioeconomic characteristics, research setting, method of measuring telomere length, reported outcome, and determinants of interest. Studies will also be stratified by 1) age into 3 groups: childhood (0 to 18 years), adulthood (19 to 64 years) and late life (65+); 2) cell type; 3) study design; and 4) telomere length assessment method. Where feasible, study results will be combined through meta-analyses to obtain a pooled measure of associations. Results will be reported according to the Preferred Reporting Items for Systematic Reviews and Meta-Analyses (PRISMA) Statement.

**Discussion:**

This systematic review will provide knowledge on the existing evidence for chronic social stress and its association with telomere lengths throughout the life course.

## Background

Stress, which the World Health Organization (WHO) calls the ‘21st-century health epidemic’ [1], generally refers to experiences that are either novel, uncontrollable, unpredictable or a threat to one’s ego. Stressful situations that originate from one’s social environment (or social stressors) include extreme poverty, violence or long-term care of a very dependent family member or friend. Social stress is common in people’s lives; in a WHO study of 21 countries, more than 10% of respondents reported witnessing violence (21.8%) or experiencing interpersonal violence (18.8%), exposure to war (16.2%) or trauma to a loved one (12.5%) [2].

The effects of stress on health-related outcomes are observed throughout the life course. Early life experiences such as physical and sexual abuse or being raised by neglectful and uncaring families impose a life-long burden of behavioral and pathophysiological problems [3,4] and long-lasting emotional problems [5]. Thus, these early life experiences have a huge impact on an individual’s response to stress. Caregiving for a disabled relative also leads to chronic stress from anxiety, poor sleep, exhaustion, depression, and sadness [6]. Also, living in extreme poverty implies exposure to severe circumstances of economic disadvantage such as hunger and lack of basic resources to cover basic needs and is associated with chronic stress [7]. Some of the effects of stress are seen on brain structure and function, and in the risk for later depression and post-traumatic stress disorder (PTSD) [8-10].

Allostasis is the active process of the body’s adaptive response to stress [11]. The body responds to almost any stressful event or challenge by rapidly releasing chemical mediators - for example, catecholamines and glucocorticoids (GCs). Pro- and anti-inflammatory cytokines are also produced by many cells in the body; they regulate each other and are, in turn, regulated by GC and catecholamines. Under acute stress, GCs inhibit the production of pro-inflammatory cytokines [12]. However, this regulation is complex and depends on the amount of circulating GC [13,14]. With chronic stress exposure, GC levels drop. Thus, pro-inflammatory cytokines are no longer inhibited, leading to long-term elevations in cytokine levels and greater risks for inflammatory diseases [15,16]. Studies on inflammatory processes after chronic life stress report a decrease in host resistance to infections [17] and higher levels of pro-inflammatory cytokines [18,19].

Numerous population studies demonstrate links between chronic stress and poor physical [15,16,20-22] and mental health [23]. The stress associated with providing care for a spouse diagnosed with Alzheimer’s disease can have adverse effects on cardiovascular health [24,25]. Also, there is a link between chronic stress and disease outcomes associated with inadequate immunity (infectious and neoplastic disease) and disease outcomes associated with excessive immune activity (allergic and autoimmune disease). The immune response becomes very dynamic in situations of chronic stress and undergoes simultaneous enhancement and suppression with altering patterns of cytokine secretion [26]. This shift can occur via the effects of stress hormones such as cortisol, the most important GC [27], and changes the balance of the immune response, resulting in greater vulnerability to autoimmune and allergic diseases; this shift in cytokine production may explain some of the stress-related changes in immune function and disease outcomes [26].

Recently, telomere shortening has been associated with psychological stress, both perceived stress and chronic stress [28]. Telomeres are repetitive DNA sequences that cap the ends of all chromosomes and protect them from deterioration. In one study, mothers of chronically ill children with high levels of perceived stress were associated with shorter telomeres equivalent to at least one decade of additional aging compared to low stress mothers with healthy children [28]. Several studies have replicated the relationship between stress and telomere length (TL) in children and in older adults [29,30]. However, one study failed to replicate the association between telomere length and physical and sexual abuse in childhood in a large cohort of adult twins [29]. The contradiction in the results across studies is puzzling.

One of the biological mechanism by which stress may affect health could be through telomere attrition. Nonetheless, not all studies are in agreement regarding the association between exposure to stress and telomere length. This association may be because it is age-dependent: younger individuals have more rapid telomere attrition rates than adults, most likely due to the higher cellular replication rate [31]. Telomere length seems to remain stable until early adulthood and gradually declines with advancing age [31]. This age-related variation in length may modify the association between TL and stress, especially when exposure to stress varies as an individual ages. Furthermore, given that individuals who are afflicted with a disease may have shorter TL, disease status may also influence the association between stress and TL. It remains unclear whether both stress and disease interact to affect the rate of telomere attrition, or whether the disease mediates the association between stress and TL. There is thus a need to explore the effects of stress on telomere length in diseased and healthy individuals.

Telomere length is a cell-specific measure; some cells have high replicative histories, while others have low ones. Other cells may express telomerase, an enzyme that lengthens telomeres and affects the length of telomeres. When examining the association between TL and stress, cell-specific telomere measurements can thus be an important issue to consider.

Many assays are currently used to estimate telomere length, each with distinct advantages and disadvantages. Depending on the assay chosen, results may vary. Some assays chosen to measure telomere length give mean telomere length for all chromosomes, others give specific values for each chromosome, and others identify relative values. The method chosen to estimate telomere length may modify the association reported between stress and TL.

Taken together, there is a strong need to evaluate systematically the existing evidence on chronic social stress and its association with telomere length throughout the life course. This information will be helpful in guiding researchers in understanding the extent of biological changes related to chronic social stress, and in discovering sources of individual variability in adverse effects of stress on telomere erosion.

### Objectives and research questions

Our objectives are to assess whether chronic social stress is associated with telomere length over the life course of human populations. To do so we will identify, assess and synthesize the literature a) to evaluate the cross-sectional association between chronic social stress and telomere length, and b) we will investigate longitudinal associations between chronic social stress and telomere length and/or the rate of telomere loss.

The research hypotheses of this systematic review are a) being exposed to chronic social stress will lead to shorter telomeres or increase the rate of telomere loss and b) the existence and strength of the association between chronic social stress and telomere length may be modified by age, disease status, tissue type and measurement method.

## Methods/Design

**Figure 1:**
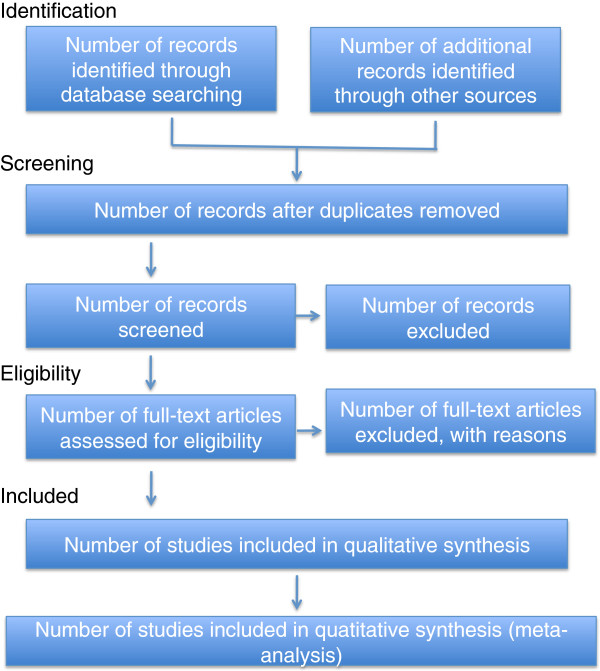
**PRISMA flow diagram.** Source: Moher *et al*. [33].

The current protocol outlines a strategy informed by the guidelines of The Cochrane Collaboration [32]. The systematic review will follow the four-phase flow diagram (Figure 1) put forth by the *P*referred *R*eporting *I*tems for *S*ystematic Reviews and *M*eta-*A*nalyses (PRISMA) Statement [33]. The methodological choices described in our protocol have been inspired by previously published work from members of our group [34]. This protocol is registered with the PROSPERO database (registration number: CRD42014009274).

### Information sources and literature search

Literature searches will be performed by the research team (JQ, MTT, and MVZ) from the *Montreal University Health Center’s Research Centre (CRCHUM*), and an expert librarian (DZ) of the *Montreal University Health Center’s documentation center (CHUM*). No language restriction will be enforced conditional to the provision of an English abstract. We will use specific medical subject headings (MeSH) and words from ‘all fields’ to identify studies in MEDLINE (PubMed interface), EMBASE (OVID interface), Cochrane Central (OVID interface), and CINAHL (EBSCO interface) from their start date onwards. We will hand-search relevant abstracts in the Cochrane Public Health Group. Details concerning the search strategy for MEDLINE, EMBASE, and Cochrane Central are provided in Additional file 1. We will search through gray literature sources, namely: System for information on Grey Literature in Europe (OpenSigle); National Guideline Clearing House; National Institutes for Health and Clinical Excellence; The Grey Literature Report (NYAM); Google Scholar and PLoS (Public Library of Science) genetics and PLoS biology. Furthermore, we will search official websites of institutions active in the fields of social stress and/or cellular aging/telomere biology, along with bibliographic references of retrieved articles and relevant reviews.

Our search strategy will combine terms related to the following themes: 1) stress; 2) telomere length; 3) life course. An additional filter for ‘humans’ will be added to the MeSH term and all fields to only include research done on human populations. All articles and reports retrieved during the identification phase will be combined in an Endnote file. Duplicates will then be extracted. Endnote entries will be further filtered by age category to examine the effects during childhood (0 to 18 years), adulthood (19 to 64 years) and late life (65 years and older). Details concerning these steps can be found in Additional files 1 and 2.

### Study inclusion criteria

#### Participants and setting

We will identify studies investigating the effects of chronic social stress in individuals with and without disease of any age (children and adults) and for both sexes.

#### Design

Our systematic review will include observational studies (cohort, case-control and cross-sectional studies) and experimental studies that investigate the impact of stress interventions on telomere length and telomere shortening.

#### Outcomes

Our primary outcomes will be telomere length, rate of telomere shortening, and estimated years lost due to telomere shortening. All methods of measuring telomere length will be considered, as well as all cell types sampled for the telomere measurements.

#### Determinants/exposures

Our main exposure will be having or experiencing chronic social stress at all ages defined as exposure to violence, poverty, or being a caregiver of a disabled person.

### Study selection procedure

A team of researchers, JQ (Epidemiologist, PhD) and MTT (Neurosciences, PhD), will identify articles by first analyzing titles and abstracts for relevance and presence of the selection criterions listed above. Inter-rater agreement in abstract screening will be computed using Cohen’s Kappa. Assuming a significance level α of 0.05 (z = 1.96), a significance level β of 0.20 (z = 0.842), κ_0_ = 70%, κ_1_ = 90%, and a positive rating of 10% per rater, we obtained a sample size of 226 abstracts [35].

Articles will be classified as i) included, ii) excluded or iii) uncertain. Records judged irrelevant will be excluded. The full text articles of selected abstracts (records categorized as included or uncertain) will be obtained for further eligibility analysis.

Full-text screening will be conducted independently by two reviewers (JQ and MTT) using a standardized form with explicit inclusion and exclusion criterions [see Additional file 3]. Discrepancies in eligibility will be resolved through discussion between the two reviewers. In the event of an unsettled disagreement, the opinion of a senior epidemiologist (MVZ) will be obtained.

### Data collection process

Reviewers will use an explicit data collection form to extract study characteristics (such as country, setting, year of publication, study design, and sample size); participants’ characteristics (age, sex, body mass index, smoking status, socioeconomic position, and health status); study exposure (type of social stress and timing of social stress); outcomes (telomere length, rate of telomere attrition, estimated years lost due to telomere shortening, method of measuring telomere length, and cell type sampled); and measures of the association between stress and telomere length with information concerning adjustments (univariate versus multivariate analyses and confounding variables). Reviewers will systematically use a standardized data abstraction form [see Additional file 3]. Reliability of data abstraction will be tested on a random sample. According to these results, and if necessary, modifications will be done to the abstraction tool. JQ and MTT will independently extract the data. Unsettled disagreements will be resolved by the senior reviewer (MVZ).

### Scientific quality assessment

The scientific quality of selected articles will be appraised. To do so, we will use standardized quality assessment tools tailored to each study design to best appraise the studies’ methodological quality and risk of bias. For randomized controlled trials (RCT), the Cochrane Collaboration’s Risk of Bias Tool (CCRBT) [32] will be utilized; for quasi-experimental designs, such as interrupted time series and controlled before-after studies, we will use the Cochrane Effective Practice and Organization of Practice (EPOC) Risk of Bias Tool [36]; for cohort, case control and cross-sectional studies, we will use the Effective Public Health Practice Project (EPHPP) Quality Assessment Tool for Quantitative Studies with extended selection bias assessment. The latter examines six domains of methodological quality including selection bias, study design, confounders, blinding, data collection methods and participant dropouts. The EPHPP grades methodological quality in each domain and then globally. Studies are then classified as being ‘strong’ - if no weak ratings are given, ‘moderate’ - if they get one weak rating, or ‘weak’ - if they obtain two or more weak ratings. The EPHPP quality tool encompasses the principal quality items identified by the *Strengthening the Reporting of Observational Studies in Epidemiology (STROBE) Statement*[37]. As suggested by Groenwold and Rovers (2010), adherence to that STROBE statement assures that the presentation of study characteristics is done in a fashion so as to facilitate its scientific appraisal [38]. The EPHPP is reported to have excellent inter-rater agreement for the final grade of studies [39,40], as well as adequate construct and content validity [41]. Special attention will be given to explicit study objectives, well-defined inclusion criteria and clear definitions of outcomes, independent factors, potential confounders and effect modifiers [42,43]. JQ and MTT will independently appraise the scientific quality of the studies. Discrepancies or uncertainties will be resolved through discussions with the senior reviewer (MVZ).

### Data synthesis

Evidence tables will be generated to descriptively summarize the included studies and results: 1) authors, 2) age group 3) cell type 4) study design, 5) objectives, 6) setting, 7) population, 8) outcome and measurement method, 9) determinants/exposures and type of stress, 10) results and 11) scientific quality. Where feasible, data will be combined to obtain pooled associative measures between stress and telomere length through meta-analyses, using The Cochrane Group’s Review Manager software (RevMan 5.1) [44]. This involves using linear regression models to analyze the association between stress and telomere length. To take account of protocol variability in blood storage, DNA extraction, and measurement method of telomere length, we will convert the absolute measures to study-specific z-scores (regression coefficients and corresponding standard errors will be divided by the standard deviation of telomere length). The cohort-specific standardized regression coefficients and standard errors will be grouped and analyzed if possible, by 1) age group (childhood (0 to 18 years), adulthood (19 to 64) and aged (65+)), 2) cell type, 3) study design, 4) telomere length measurement method, and 5) type of stress. Because the sources of, and tolerance to, chronic social stress may be different in lower- and middle-income countries as compared to high-income countries, we will also stratify (if possible) participants based on studies originating from each of these settings. We will use The World Bank Group’s classification to identify those countries [see Additional file 4] [45]. Due consideration will be given to heterogeneity. We will first qualitatively examine heterogeneity in the study designs, settings, populations and exposure/outcome definitions and measurements. We will then quantitatively assess heterogeneity via Cochran’s Q Test with a liberal significance level [46], and quantify such heterogeneity via the *I*^2^ statistic using Higgins *et al*.’s (2003) classification [47]. Due consideration will be provided to possible outlier results in the effect estimates by visually inspecting forest plots. Where feasible, we will carry out separate random effects meta-analyses [48] of adjusted versus non-adjusted (or insufficiently adjusted) standardized linear regression association measures between stress and TL. If conditions impede meta-analyses, data will be synthesized narratively. Results will be reported according to the *P*referred *R*eporting *I*tems for *S*ystematic Reviews and *M*eta-*A*nalyses (PRISMA) Statement [33], and measures of variation, such as 95% confidence intervals and *P* values for each estimate will be provided. To assess the possible influence of publication bias on the results, funnel plots and statistical tests to measure the extent of this bias will be performed [49].

## Discussion

This systematic review will provide:

1. Knowledge on the existing evidence for chronic social stress and its association with telomere length throughout the different stages of the life course (childhood, adulthood, and late life).

2. Pragmatic reasons on why health policy planners need to prioritize stress reduction at the societal level, including childhood poverty eradication, income security supplements in old age and caregivers respite care, and at the individual level, including physical activity and individual stress management techniques as possible prevention of TL erosion.

3. An overview of knowledge gaps and future research needs.

Importantly, this review will provide evidence for the embodiment of stress and its impact on population premature aging and early onset of chronic disease, emphasizing the importance of developing novel coping resources that may protect individuals from the adverse effects of stress on telomere erosion as primary future directions in this field. Given that individuals who are exposed to stress during their early years show a faster erosion rate of TL, early intervention and prevention strategies can potentially ameliorate the acceleration of physiological aging processes. It is also hoped that the knowledge from this review will be used to direct future research in testing TL as a marker of outcomes of stress intervention programs (such as psychological or physiotherapy programs), or public policies (such as free child care centers, respite services, *etcetera*). Finally, it is hoped that the results from this review will push future research in identifying novel targets (that act to maintain or elongate telomeres, for example) for intervention to help individuals recover from exposure to stress. The results from this review are limited by study design. Longitudinal findings on TL indicate that results should be interpreted with caution since the temporal process of telomere erosion is complex. In addition, use of different telomere length measurement methods and/or tissue types may limit valid comparisons between studies. Results of the systematic review will be published in a peer-reviewed international journal and presented at conferences and symposia in relevant fields. Further knowledge dissemination will involve communicating results to the governments and to organizations active in promoting stress reduction programs. The utmost relevance of systematic reviews to inform health policymaking is increasingly recognized (48). Our review will supply evidence to health policy planners on the importance of poverty and violence reduction programs and caregiving respite care for this generation, and future generations.

## Abbreviations

GC: glucocorticoids; TL: telomere length; WHO: World Health Organization.

## Competing interests

The authors declare that they have no competing interests.

## Authors’ contributions

JQ, MTT, HF, MK, EVL and MVZ contributed to the conception and design of the review. JQ, MTT, MVZ, and DZ developed the search strategies. JQ drafted the manuscript with the help of EVL and MTT. MTT and MVZ were actively involved in critically revising the protocol for important intellectual content. DZ made a substantial contribution to the Information sources and literature search section, and to Additional file 1. All authors read and approved the final manuscript.

## Supplementary Material

Additional file 1 Search strategy MEDLINE, EMBASE, Cochrane Central, and CINAHL. Click here for file

Additional file 2 Endnote file manipulations. Click here for file

Additional file 3 Data collection form. Click here for file

Additional file 4 The World Bank Group’s classification of countries by income groups. Click here for file
