# Supplementary material for: Protocol for a systematic review of the association between chronic stress during the life course and telomere length
Source: Syst Rev. 2014 Apr 30;3:40. doi: 10.1186/2046-4053-3-40 (PMC4022427; doi:10.1186/2046-4053-3-40)
Supplement: Additional file 1 — Search strategy MEDLINE, EMBASE, Cochrane Central, and CINAHL. [file 2046-4053-3-40-S1.docx]

**Additional file 1.** **Search Strategy** *- Protocol for a systematic review on the association between chronic social stress and telomere length.*

**1. MEDLINE (PubMed interface)**

((((((((((((((((((((((((((((((((((((((((((((((((((("Stress"[Journal] OR "stress"[All Fields]) OR stresses[All Fields]) OR stressor[All Fields]) OR stressors[All Fields]) OR adversity[All Fields]) OR adversities[All Fields]) OR ("poverty"[MeSH Terms] OR "poverty"[All Fields])) OR ("poverty"[MeSH Terms] OR "poverty"[All Fields] OR "poor"[All Fields])) OR ("hunger"[MeSH Terms] OR "hunger"[All Fields])) OR ("hunger"[MeSH Terms] OR "hunger"[All Fields] OR "hungry"[All Fields])) OR (("economics"[MeSH Terms] OR "economics"[All Fields] OR "economic"[All Fields]) AND insecurity[All Fields])) OR ("unemployment"[MeSH Terms] OR "unemployment"[All Fields])) OR ("unemployment"[MeSH Terms] OR "unemployment"[All Fields] OR "unemployed"[All Fields])) OR ("homeless persons"[MeSH Terms] OR ("homeless"[All Fields] AND "persons"[All Fields]) OR "homeless persons"[All Fields] OR "homeless"[All Fields])) OR ("social welfare"[MeSH Terms] OR ("social"[All Fields] AND "welfare"[All Fields]) OR "social welfare"[All Fields])) OR ("vulnerable populations"[MeSH Terms] OR ("vulnerable"[All Fields] AND "populations"[All Fields]) OR "vulnerable populations"[All Fields] OR "disadvantaged"[All Fields])) OR ("violence"[MeSH Terms] OR "violence"[All Fields])) OR ("conflict (psychology)"[MeSH Terms] OR ("conflict"[All Fields] AND "(psychology)"[All Fields]) OR "conflict (psychology)"[All Fields] OR "conflict"[All Fields])) OR ("refugees"[MeSH Terms] OR "refugees"[All Fields] OR "refugee"[All Fields])) OR ("refugees"[MeSH Terms] OR "refugees"[All Fields])) OR ("war"[MeSH Terms] OR "war"[All Fields])) OR victim[All Fields]) OR victims[All Fields]) OR (("holocaust"[MeSH Terms] OR "holocaust"[All Fields]) AND ("survivors"[MeSH Terms] OR "survivors"[All Fields] OR "survivor"[All Fields]))) OR (("holocaust"[MeSH Terms] OR "holocaust"[All Fields]) AND ("survivors"[MeSH Terms] OR "survivors"[All Fields]))) OR tension[All Fields]) OR ("injuries"[Subheading] OR "injuries"[All Fields] OR "trauma"[All Fields] OR "wounds and injuries"[MeSH Terms] OR ("wounds"[All Fields] AND "injuries"[All Fields]) OR "wounds and injuries"[All Fields])) OR traumatic[All Fields]) OR distress[All Fields]) OR (conjugal[All Fields] AND ("violence"[MeSH Terms] OR "violence"[All Fields]))) OR ("family conflict"[MeSH Terms] OR ("family"[All Fields] AND "conflict"[All Fields]) OR "family conflict"[All Fields] OR ("marital"[All Fields] AND "conflict"[All Fields]) OR "marital conflict"[All Fields])) OR (troubled[All Fields] AND relationships[All Fields])) OR (("sprains and strains"[MeSH Terms] OR ("sprains"[All Fields] AND "strains"[All Fields]) OR "sprains and strains"[All Fields] OR "strained"[All Fields]) AND relationships[All Fields])) OR ("divorce"[MeSH Terms] OR "divorce"[All Fields])) OR maltreatment[All Fields]) OR (relationships[All Fields] AND ("conflict (psychology)"[MeSH Terms] OR ("conflict"[All Fields] AND "(psychology)"[All Fields]) OR "conflict (psychology)"[All Fields] OR "conflict"[All Fields]))) OR ("substance-related disorders"[MeSH Terms] OR ("substance-related"[All Fields] AND "disorders"[All Fields]) OR "substance-related disorders"[All Fields] OR "abuse"[All Fields])) OR (("child"[MeSH Terms] OR "child"[All Fields] OR "children"[All Fields]) AND exposed[All Fields] AND ("ethanol"[MeSH Terms] OR "ethanol"[All Fields] OR "alcohol"[All Fields] OR "alcohols"[MeSH Terms] OR "alcohols"[All Fields]))) OR (("child"[MeSH Terms] OR "child"[All Fields] OR "children"[All Fields]) AND exposed[All Fields] AND ("pharmaceutical preparations"[MeSH Terms] OR ("pharmaceutical"[All Fields] AND "preparations"[All Fields]) OR "pharmaceutical preparations"[All Fields] OR "drugs"[All Fields]))) OR (("depressive disorder"[MeSH Terms] OR ("depressive"[All Fields] AND "disorder"[All Fields]) OR "depressive disorder"[All Fields] OR "depression"[All Fields] OR "depression"[MeSH Terms]) AND ("pregnancy"[MeSH Terms] OR "pregnancy"[All Fields]))) OR (("child"[MeSH Terms] OR "child"[All Fields] OR "children"[All Fields]) AND exposed[All Fields] AND ("depressive disorder"[MeSH Terms] OR ("depressive"[All Fields] AND "disorder"[All Fields]) OR "depressive disorder"[All Fields] OR "depression"[All Fields] OR "depression"[MeSH Terms]))) OR ("depression, postpartum"[MeSH Terms] OR ("depression"[All Fields] AND "postpartum"[All Fields]) OR "postpartum depression"[All Fields] OR ("post"[All Fields] AND "partum"[All Fields] AND "depression"[All Fields]) OR "post partum depression"[All Fields])) OR ("substance-related disorders"[MeSH Terms] OR ("substance-related"[All Fields] AND "disorders"[All Fields]) OR "substance-related disorders"[All Fields] OR "abuse"[All Fields])) OR ("caregivers"[MeSH Terms] OR "caregivers"[All Fields] OR ("care"[All Fields] AND "givers"[All Fields]) OR "care givers"[All Fields])) OR (care[All Fields] AND giving[All Fields])) OR ("caregivers"[MeSH Terms] OR "caregivers"[All Fields] OR ("care"[All Fields] AND "giver"[All Fields]) OR "care giver"[All Fields])) OR ("caregivers"[MeSH Terms] OR "caregivers"[All Fields] OR ("care"[All Fields] AND "givers"[All Fields]) OR "care givers"[All Fields])) OR care-giving[All Fields]) OR ("caregivers"[MeSH Terms] OR "caregivers"[All Fields] OR "caregiver"[All Fields])) OR ("caregivers"[MeSH Terms] OR "caregivers"[All Fields])) OR caregiving[All Fields])

AND

((((((((("telomere"[MeSH Terms] OR "telomere"[All Fields]) OR ("cell aging"[MeSH Terms] OR ("cell"[All Fields] AND "aging"[All Fields]) OR "cell aging"[All Fields] OR ("cellular"[All Fields] AND "aging"[All Fields]) OR "cellular aging"[All Fields])) OR ("cell aging"[MeSH Terms] OR ("cell"[All Fields] AND "aging"[All Fields]) OR "cell aging"[All Fields])) OR (("leukocytes"[MeSH Terms] OR "leukocytes"[All Fields] OR "leukocyte"[All Fields]) AND ("telomere"[MeSH Terms] OR "telomere"[All Fields]) AND length[All Fields])) OR LTL[All Fields]) OR ("cell aging"[MeSH Terms] OR ("cell"[All Fields] AND "aging"[All Fields]) OR "cell aging"[All Fields] OR ("cellular"[All Fields] AND "senescence"[All Fields]) OR "cellular senescence"[All Fields])) OR ("cell aging"[MeSH Terms] OR ("cell"[All Fields] AND "aging"[All Fields]) OR "cell aging"[All Fields] OR ("replicative"[All Fields] AND "senescence"[All Fields]) OR "replicative senescence"[All Fields])) OR ("cell aging"[MeSH Terms] OR ("cell"[All Fields] AND "aging"[All Fields]) OR "cell aging"[All Fields] OR ("cell"[All Fields] AND "senescence"[All Fields]) OR "cell senescence"[All Fields])) OR telome[All Fields])

AND the age filters

for « 65 + years » :

("aged"[MeSH Terms] OR "aged, 80 and over"[MeSH Terms] OR (("aged"[MeSH Terms] OR "aged"[All Fields]) AND 65[All Fields] AND years[All Fields] AND over[All Fields]) OR "aged, 80 and over"[All Fields]) AND ("aged"[MeSH Terms] OR "aged, 80 and over"[MeSH Terms]) OR (65[All Fields] AND years[All Fields] AND over[All Fields]) OR "65 years"[All Fields] OR "66 years"[All Fields] OR "67 years"[All Fields] OR "68 years"[All Fields] OR "69 years"[All Fields] OR "70 years"[All Fields] OR "71 years"[All Fields] OR "72 years"[All Fields] OR "73 years"[All Fields] OR "74 years"[All Fields] OR "75 years"[All Fields] OR "76 years"[All Fields] OR "77 years"[All Fields] OR "78 years"[All Fields] OR "79 years"[All Fields] OR "80 years"[All Fields] OR "81 years"[All Fields] OR "82 years"[All Fields] OR "83 years"[All Fields] OR "84 years"[All Fields] OR "85 years"[All Fields] OR "86 years"[All Fields] OR "87 years"[All Fields] OR "88 years"[All Fields] OR "89 years"[All Fields] OR "90 years"[All Fields] OR "91 years"[All Fields] OR "92 years"[All Fields] OR "93 years"[All Fields] OR "94 years"[All Fields] OR "95 years"[All Fields] OR "96 years"[All Fields] OR "97 years"[All Fields] OR "98 years"[All Fields] OR "99 years"[All Fields] OR "100 years"[All Fields] OR ("aged"[MeSH Terms] OR "aged"[All Fields] OR "elderly"[All Fields]) OR senior[All Fields] OR ("aged"[MeSH Terms] OR "aged"[All Fields]) OR ("sambucus"[MeSH Terms] OR "sambucus"[All Fields] OR "elder"[All Fields]) OR "late life"[All Fields]

For « 0 - 18 years » :

"infant"[MeSH Terms] OR "child"[MeSH Terms] OR "adolescent"[MeSH Terms] OR "infant"[All Fields] OR "child"[All Fields] OR "adolescent"[All Fields] OR "1 year"[All Fields] OR "2 years"[All Fields] OR "3 years"[All Fields] OR "5 years"[All Fields] OR "6 years"[All Fields] OR "7 years"[All Fields] OR "8 years"[All Fields] OR "9 years"[All Fields] OR "10 years"[All Fields] OR "11 years"[All Fields] OR "12 years"[All Fields] OR "13 years"[All Fields] OR "14 years"[All Fields] OR "15 years"[All Fields] OR "16 years"[All Fields] OR "17 years"[All Fields] OR "18 years"[All Fields]

For « 19 - 64 years »

"adult"[MeSH Terms:noexp] OR "middle aged"[MeSH Terms] OR "19 years"[All Fields] OR "middle aged"[All Fields] OR "20 years"[All Fields] OR "21 years"[All Fields] OR "22 years"[All Fields] OR "23 years"[All Fields] OR "24 years"[All Fields] OR "25 years"[All Fields] OR "26 years"[All Fields] OR "27 years"[All Fields] OR "28 years"[All Fields] OR "29 years"[All Fields] OR "30 years"[All Fields] OR "31 years"[All Fields] OR "32 years"[All Fields] OR "33 years"[All Fields] OR "34 years"[All Fields] OR "35 years"[All Fields] OR "36 years"[All Fields] OR "37 years"[All Fields] OR "38 years"[All Fields] OR "39 years"[All Fields] OR "40 years"[All Fields] OR "41 years"[All Fields] OR "42 years"[All Fields] OR "43 years"[All Fields] OR "44 years"[All Fields] OR "45 years"[All Fields] OR "46 years"[All Fields] OR "47 years"[All Fields] OR "48 years"[All Fields] OR "49 years"[All Fields] OR "50 years"[All Fields] OR "51 years"[All Fields] OR "52 years"[All Fields] OR "53 years"[All Fields] OR "54 years"[All Fields] OR "55 years"[All Fields] OR "56 years"[All Fields] OR "57 years"[All Fields] OR "58 years"[All Fields] OR "59 years"[All Fields] OR "60 years"[All Fields] OR "61 years"[All Fields] OR "62 years"[All Fields] OR "63 years"[All Fields] OR "64 yea

**2. EMBASE (OVID interface)**

**For 0-18 years:**

Database: Embase <1974 to 2013 Week 43>

Search Strategy:

--------------------------------------------------------------------------------

1 telomer*.af. (34515)

2 cellular aging.af. (1124)

3 chromosome* end*.af. (1655)

4 cell aging.af. (11447)

5 chromosome* cap*.af. (105)

6 leukocyte* telomere* length.af. (344)

7 LTL.af. (346)

8 tert.af. (42111)

9 terc.af. (946)

10 chromatid* end*.af. (11)

11 replicative senescence.af. (1712)

12 cellular senescence.af. (4104)

13 cell senescence.af. (1701)

14 telome*.af. (34590)

15 or/1-14 (88331)

16 Stress*.af. (806589)

17 adversit*.af. (4045)

18 povert*.af. (37212)

19 poor.af. (431300)

20 hunger.af. (11451)

21 hungry.af. (1875)

22 economic insecurit*.af. (64)

23 unemployment.af. (12943)

24 unemployed.af. (6612)

25 homeless.af. (6113)

26 social welfare.af. (19393)

27 disadvantaged.af. (8342)

28 violence.af. (55483)

29 conflict.af. (57862)

30 refugee*.af. (9340)

31 war*.af. (744041)

32 victim*.af. (47966)

33 Holocaust survivor*.af. (408)

34 tension*.af. (144861)

35 distress.af. (127498)

36 marital conflict*.af. (641)

37 troubled relationship*.af. (46)

38 strained relationship*.af. (65)

39 divorce*.af. (10046)

40 maltreatment*.af. (4343)

41 "relationship* conflict*".af. (151)

42 "children exposed to alcohol".af. (58)

43 "children exposed to drugs".af. (10)

44 "depression during pregnancy".af. (516)

45 "children exposed to depression".af. (0)

46 "children exposed to depression*".af. (0)

47 post-partum depression*.af. (256)

48 abuse*.af. (208715)

49 care giver*.af. (2922)

50 care giving.af. (1235)

51 care-giver*.af. (2922)

52 care-giving.af. (1235)

53 caregiver*.af. (54209)

54 caregiving.af. (7350)

55 or/16-54 (2527423)

56 and/15,55 (11784)

57 limit 56 to embase (10201)

58 limit 57 to human (6086)

59 limit 58 to (infant <to one year> or child <unspecified age> or preschool child <1 to 6 years> or school child <7 to 12 years> or adolescent <13 to 17 years>) (238)

***************************

**For 19-64 years:**

Database: Embase <1974 to 2013 Week 43>

Search Strategy:

--------------------------------------------------------------------------------

1 telomer*.af. (34515)

2 cellular aging.af. (1124)

3 chromosome* end*.af. (1655)

4 cell aging.af. (11447)

5 chromosome* cap*.af. (105)

6 leukocyte* telomere* length.af. (344)

7 LTL.af. (346)

8 tert.af. (42111)

9 terc.af. (946)

10 chromatid* end*.af. (11)

11 replicative senescence.af. (1712)

12 cellular senescence.af. (4104)

13 cell senescence.af. (1701)

14 telome*.af. (34590)

15 or/1-14 (88331)

16 Stress*.af. (806589)

17 adversit*.af. (4045)

18 povert*.af. (37212)

19 poor.af. (431300)

20 hunger.af. (11451)

21 hungry.af. (1875)

22 economic insecurit*.af. (64)

23 unemployment.af. (12943)

24 unemployed.af. (6612)

25 homeless.af. (6113)

26 social welfare.af. (19393)

27 disadvantaged.af. (8342)

28 violence.af. (55483)

29 conflict.af. (57862)

30 refugee*.af. (9340)

31 war*.af. (744041)

32 victim*.af. (47966)

33 Holocaust survivor*.af. (408)

34 tension*.af. (144861)

35 distress.af. (127498)

36 marital conflict*.af. (641)

37 troubled relationship*.af. (46)

38 strained relationship*.af. (65)

39 divorce*.af. (10046)

40 maltreatment*.af. (4343)

41 "relationship* conflict*".af. (151)

42 "children exposed to alcohol".af. (58)

43 "children exposed to drugs".af. (10)

44 "depression during pregnancy".af. (516)

45 "children exposed to depression".af. (0)

46 "children exposed to depression*".af. (0)

47 post-partum depression*.af. (256)

48 abuse*.af. (208715)

49 care giver*.af. (2922)

50 care giving.af. (1235)

51 care-giver*.af. (2922)

52 care-giving.af. (1235)

53 caregiver*.af. (54209)

54 caregiving.af. (7350)

55 or/16-54 (2527423)

56 and/15,55 (11784)

57 limit 56 to embase (10201)

58 limit 57 to human (6086)

59 limit 58 to adult <18 to 64 years> (801)

***************************

**For 65+ years:**

Database: Embase <1974 to 2013 Week 43>

Search Strategy:

--------------------------------------------------------------------------------

1 telomer*.af. (34515)

2 cellular aging.af. (1124)

3 chromosome* end*.af. (1655)

4 cell aging.af. (11447)

5 chromosome* cap*.af. (105)

6 leukocyte* telomere* length.af. (344)

7 LTL.af. (346)

8 tert.af. (42111)

9 terc.af. (946)

10 chromatid* end*.af. (11)

11 replicative senescence.af. (1712)

12 cellular senescence.af. (4104)

13 cell senescence.af. (1701)

14 telome*.af. (34590)

15 or/1-14 (88331)

16 Stress*.af. (806589)

17 adversit*.af. (4045)

18 povert*.af. (37212)

19 poor.af. (431300)

20 hunger.af. (11451)

21 hungry.af. (1875)

22 economic insecurit*.af. (64)

23 unemployment.af. (12943)

24 unemployed.af. (6612)

25 homeless.af. (6113)

26 social welfare.af. (19393)

27 disadvantaged.af. (8342)

28 violence.af. (55483)

29 conflict.af. (57862)

30 refugee*.af. (9340)

31 war*.af. (744041)

32 victim*.af. (47966)

33 Holocaust survivor*.af. (408)

34 tension*.af. (144861)

35 distress.af. (127498)

36 marital conflict*.af. (641)

37 troubled relationship*.af. (46)

38 strained relationship*.af. (65)

39 divorce*.af. (10046)

40 maltreatment*.af. (4343)

41 "relationship* conflict*".af. (151)

42 "children exposed to alcohol".af. (58)

43 "children exposed to drugs".af. (10)

44 "depression during pregnancy".af. (516)

45 "children exposed to depression".af. (0)

46 "children exposed to depression*".af. (0)

47 post-partum depression*.af. (256)

48 abuse*.af. (208715)

49 care giver*.af. (2922)

50 care giving.af. (1235)

51 care-giver*.af. (2922)

52 care-giving.af. (1235)

53 caregiver*.af. (54209)

54 caregiving.af. (7350)

55 or/16-54 (2527423)

56 and/15,55 (11784)

57 limit 56 to (human and aged <65+ years>) (629)

58 limit 57 to embase (516)

***************************

**3. Cochrane Central (OVID interface)**

**Database: EBM Reviews - Cochrane Database of Systematic Reviews <2005 to September 2013>, EBM Reviews - ACP Journal Club <1991 to October 2013>, EBM Reviews - Database of Abstracts of Reviews of Effects <3rd Quarter 2013>, EBM Reviews - Cochrane Central Register of Controlled Trials <September 2013>, EBM Reviews - Cochrane Methodology Register <3rd Quarter 2012>, EBM Reviews - Health Technology Assessment <3rd Quarter 2013>, EBM Reviews - NHS Economic Evaluation Database <3rd Quarter 2013>**

**Search Strategy:**

**--------------------------------------------------------------------------------**

**1 telomer*.af. (78)**

**2 cellular aging.af. (2)**

**3 chromosome* end*.af. (0)**

**4 cell aging.af. (25)**

**5 chromosome* cap*.af. (0)**

**6 leukocyte* telomere* length.af. (2)**

**7 LTL.af. (8)**

**8 tert.af. (77)**

**9 terc.af. (1)**

**10 chromatid* end*.af. (0)**

**11 replicative senescence.af. (0)**

**12 cellular senescence.af. (0)**

**13 cell senescence.af. (2)**

**14 telome*.af. (78)**

**15 or/1-14 (187)**

**16 Stress*.af. (21901)**

**17 adversit*.af. (74)**

**18 povert*.af. (1262)**

**19 poor.af. (19862)**

**20 hunger.af. (988)**

**21 hungry.af. (89)**

**22 economic insecurit*.af. (0)**

**23 unemployment.af. (274)**

**24 unemployed.af. (289)**

**25 homeless.af. (422)**

**26 social welfare.af. (231)**

**27 disadvantaged.af. (629)**

**28 violence.af. (1197)**

**29 conflict.af. (1987)**

**30 refugee*.af. (182)**

**31 war*.af. (27037)**

**32 victim*.af. (890)**

**33 Holocaust survivor*.af. (1)**

**34 tension*.af. (4802)**

**35 distress.af. (7443)**

**36 marital conflict*.af. (17)**

**37 troubled relationship*.af. (0)**

**38 strained relationship*.af. (1)**

**39 divorce*.af. (186)**

**40 maltreatment*.af. (142)**

**41 "relationship* conflict*".af. (11)**

**42 "children exposed to alcohol".af. (3)**

**43 "children exposed to drugs".af. (0)**

**44 "depression during pregnancy".af. (41)**

**45 "children exposed to depression".af. (0)**

**46 "children exposed to depression*".af. (0)**

**47 post-partum depression*.af. (29)**

**48 abuse*.af. (6392)**

**49 care giver*.af. (338)**

**50 care giving.af. (78284)**

**51 care-giver*.af. (338)**

**52 care-giving.af. (78284)**

**53 caregiver*.af. (3920)**

**54 caregiving.af. (365)**

**55 or/16-54 (140881)**

**56 and/15,55 (43)**

*******************************

**4. CINAHL**

**
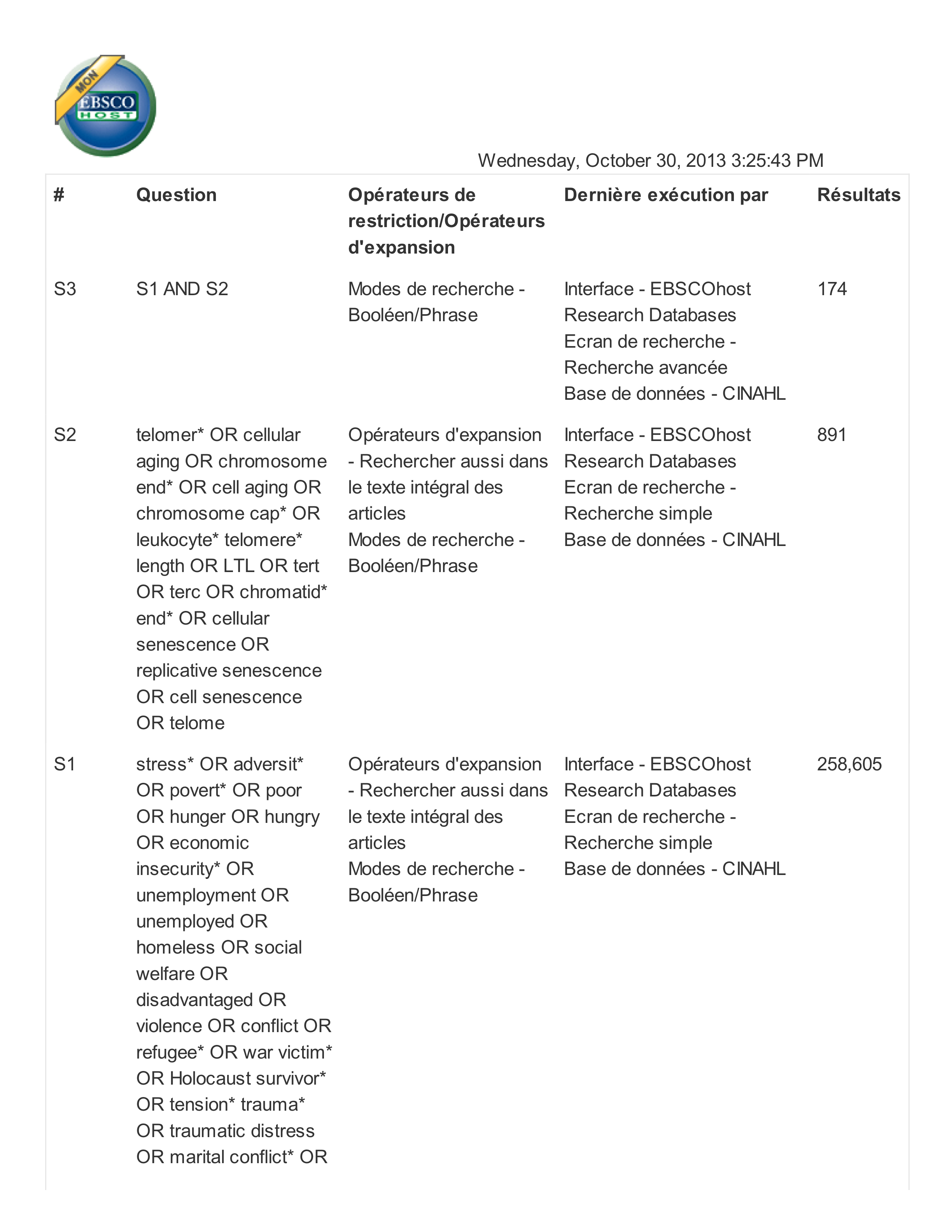
**
