# Supplementary material for: Protocol for a systematic review of the association between chronic stress during the life course and telomere length
Source: Syst Rev. 2014 Apr 30;3:40. doi: 10.1186/2046-4053-3-40 (PMC4022427; doi:10.1186/2046-4053-3-40)
Supplement: Additional file 2 — Endnote file manipulations. [file 2046-4053-3-40-S2.docx]

**Additional file 2.** **Endnote file manipulations** *- Protocol for a systematic review on the association between chronic social stress and telomere length*

1. Entries uncovered by search strategies will be combined in an Endnote file
2. Duplicates will be removed
3. Filters based on age will be applied: childhood (0-18 years), adult (19-64) and aged (65+)
4. Research on non-humans will be removed
