# Supplementary material for: Protocol for a systematic review of the association between chronic stress during the life course and telomere length
Source: Syst Rev. 2014 Apr 30;3:40. doi: 10.1186/2046-4053-3-40 (PMC4022427; doi:10.1186/2046-4053-3-40)
Supplement: Additional file 3 — Data collection form. [file 2046-4053-3-40-S3.docx]

**Additional file 3.**

Data collection form

## Notes:

- Be consistent in the order and style used to describe each report.
- Record any missing or unclear information so to indicate that the information was not found in the study report(s) but not forgotten.

| Review title | Systematic Review on the association between chronic social stress and telomere length |
| --- | --- |
| Study ID *(surname of first author and year first full report of study was published e.g. Smith 2001)* |  |
| Notes | |

# General Information

| Date form completed *(dd/mm/yyyy)* |  |
| --- | --- |
| Name/ID of person extracting data |  |
| Reference citation (e.g. Medline) |  |
| Study author contact details |  |
| Publication type  *(e.g. full report, abstract, letter)* |  |
| Notes: | |

# Study eligibility

| Study Characteristics | Eligibility criteria | | Eligibility criteria met? | | | Location in text or source *(pg & ¶/fig/table)* |
| --- | --- | --- | --- | --- | --- | --- |
|  |  | | **Yes** | **No** | **Unclear** |  |
| Type of study | Observational studies including cohort, case-control and cross-sectional studies or experimental study | |  |  |  |  |
| Participants and setting | Child, adult, aged population, from low, middle or high income country as listed in the *World Bank Group’s classification of countries by income groups (see Annexe)*  Healthy or diseased population | |  |  |  |  |
| Types of stressors exposed to | Violence  Poverty  Caregiver | |  |  |  |  |
| Types of outcome measures | **Primary outcome:**   1. Telomere length 2. Years lost due to telomere attrition 3. Cell type specified | |  |  |  |  |
| Results | Associative measure between stress and telomere length | |  |  |  |  |
| INCLUDE | EXCLUDE | |  |  |  |  |
| Reason for exclusion | |  | | | | |
| Notes: |  | | | | | |
|  | | | | | | |

**DO NOT PROCEED IF STUDY EXCLUDED FROM REVIEW**

# Characteristics of included studies

## Methods

|  | **Descriptions as stated in report/paper** | | **Location in text or source** *(pg & ¶/fig/table)* |
| --- | --- | --- | --- |
| **Aim of study** |  | |  |
| **Design** |  | |  |
| **Unit of observation** |  | |  |
| **Start date** |  | |  |
| **End date** |  | |  |
| **Duration of participation**  *(from recruitment to last follow-up)* |  | |  |
| **Ethical approval needed/ obtained for study** | Yes No Unclear |  |  |
| **Notes:** | | | |

## Participants and immune response

|  | Description | | Location in text or source *(pg & ¶/fig/table)* |
| --- | --- | --- | --- |
| Population description  *(from which study participants were drawn)* |  | |  |
| Setting and context |  | |  |
| Inclusion criterions |  | |  |
| Exclusion criterions |  | |  |
| Method of recruitment of participants *(e.g. phone, mail, clinic patients)* |  | |  |
| Informed consent obtained | Yes No Unclear |  |  |
| Total no. of subjects |  | |  |
| Participation agreement (%) |  | |  |
| Clusters  *(if applicable, no., type, no. people per cluster)* |  | |  |
| Baseline discrepancies  *(if applicable)* |  | |  |
| Lost to follow-up, mortality, withdrawals and exclusions |  | |  |
| Number of total person-years (if applicable) |  | |  |
| Missing data |  | |  |
| **Outcome(s)**  Definition, measure & classification | Primary outcome  1) Telomere length  2) Telomere attrition rate  3) Number of years lost  *Indicate if used as continuous or categorized (indicate cut-off points used by the authors): __________________________________________________________________________________________________________________________________________________________________*  Method used to measure telomere length:  1) relatice qPCR 🞏  2) absolute qPCR 🞏  3) RFLP 🞏  4) FISH 🞏  5) Other 🞏  ____________________________________________________________________________________________________________ | |  |
|  | Secondary outcomes  1) Telomere attrtion rate  2) Number of years lost  Operational definition of attrition rate or years lost used by the authors: ________________________________________________________________________________________________________________________________________________________________________________________________________________________ | |  |
| **Confounding factors/ effect modifiers** accounted for in the analyses  **Results**  *(specify, e.g. OR, RR, IRR)*  *(specify the reference group)*  **Reported limitations** of study’s methods/results |  | |  |
|  | **Crude** | |  |
|  | **Adjusted** | |  |
|  |  | |  |
| **Key conclusions** *(as stated in report/paper)*  **Scientific quality** (specify tool)  authors  Notes: |  | |  |
|  |  | |  |

## Other information

| Study funding sources  *(including role of funders)* |  |  |
| --- | --- | --- |
| Possible conflicts of interest  *(for study authors)* |  |  |
| References of relevant studies |  |  |
| Correspondence required for further study information *(from whom, what and when)* |  | |
| Notes: | | |
