# Supplementary material for: Protocol for a systematic review of the association between chronic stress during the life course and telomere length
Source: Syst Rev. 2014 Apr 30;3:40. doi: 10.1186/2046-4053-3-40 (PMC4022427; doi:10.1186/2046-4053-3-40)
Supplement: Additional file 4 — The World Bank Group’s classification of countries by income groups. [file 2046-4053-3-40-S4.docx]

**Additional file 4**

*The World Bank Group’s classification of countries by income groups*

Available from:

<http://data.worldbank.org/about/country-classifications/country-and-lending-groups>

**Low-income economies**

| Afghanistan | Gambia, The | Mozambique |
| --- | --- | --- |
| Bangladesh | Guinea | Myanmar |
| Benin | Guinea-Bisau | Nepal |
| Burkina Faso | Haiti | Niger |
| Burundi | Kenya | Rwanda |
| Cambodia | Korea, Dem Rep. | Sierra Leone |
| Central African Republic | Kyrgyz Republic | Somalia |
| Chad | Liberia | Tajikistan |
| Comoros | Madagascar | Tanzania |
| Congo, Dem. Rep | Malawi | Togo |
| Eritrea | Mali | Uganda |
| Ethiopia | Mauritania | Zimbabwe |

**Lower-middle-income economies**

| Albania | Indonesia | Samoa |
| --- | --- | --- |
| Armenia | India | São Tomé and Principe |
| Belize | Iraq | Senegal |
| Bhutan | Kiribati | Solomon Islands |
| Bolivia | Kosovo | South Sudan |
| Cameroon | Lao PDR | Sri Lanka |
| Cape Verde | Lesotho | Sudan |
| Congo, Rep. | Marshall Islands | Swaziland |
| Côte d'Ivoire | Micronesia, Fed. Sts. | Syrian Arab Republic |
| Djibouti | Moldova | Timor-Leste |
| Egypt, Arab Rep. | Mongolia | Tonga |
| El Salvador | Morocco | Ukraine |
| Fiji | Nicaragua | Uzbekistan |
| Georgia | Nigeria | Vanuatu |
| Ghana | Pakistan | Vietnam |
| Guatemala | Papua New Guinea | West Bank and Gaza |
| Guyana | Paraguay | Yemen, Rep. |
| Honduras | Philippines | Zambia |

**Upper-middle-income economies**

| Angola | Ecuador | Palau |
| --- | --- | --- |
| Algeria | Gabon | Panama |
| American Samoa | Grenada | Peru |
| Antigua and Barbuda | Iran, Islamic Rep. | Romania |
| Argentina | Jamaica | Russian Federation |
| Azerbaijan | Jordan | Serbia |
| Belarus | Kazakhstan | Seychelles |
| Bosnia and Herzegovina | Latvia | South Africa |
| Botswana | Lebanon | St. Lucia |
| Brazil | Libya | St. Vincent and the Grenadines |
| Bulgaria | Lithuania | Suriname |
| Chile | Macedonia, FYR | Thailand |
| China | Malaysia | Tunisia |
| Colombia | Maldives | Turkey |
| Costa Rica | Mauritius | Turkmenistan |
| Cuba | Mexico | Tuvalu |
| Dominica | Montenegro | Uruguay |
| Dominican Republic | Namibia | Venezuela, RB |
